# Effects of Proof Mass Geometry on Piezoelectric Vibration Energy Harvesters

**DOI:** 10.3390/s18051584

**Published:** 2018-05-16

**Authors:** Abdul Hafiz Alameh, Mathieu Gratuze, Mohannad Y. Elsayed, Frederic Nabki

**Affiliations:** Department of Electrical Engineering, École de Technologie Supérieure, Montréal, QC H3C 1K3, Canada; abdul-hafiz.alameh.1@ens.etsmtl.ca (A.H.A.); mathieu.gratuze.1@ens.etsmtl.ca (M.G.); mohannad.elsayed@mail.mcgill.ca (M.Y.E.)

**Keywords:** energy harvester, piezoelectric transducer, geometry, proof mass, resonant frequency

## Abstract

Piezoelectric energy harvesters have proven to have the potential to be a power source in a wide range of applications. As the harvester dimensions scale down, the resonance frequencies of these devices increase drastically. Proof masses are essential in micro-scale devices in order to decrease the resonance frequency and increase the strain along the beam to increase the output power. In this work, the effects of proof mass geometry on piezoelectric energy harvesters are studied. Different geometrical dimension ratios have significant impact on the resonance frequency, e.g., beam to mass lengths, and beam to mass widths. A piezoelectric energy harvester has been fabricated and tested operating at a frequency of about 4 kHz within the audible range. The responses of various prototypes were studied, and an optimized T-shaped piezoelectric vibration energy harvester design is presented for improved performance.

## 1. Introduction

Piezoelectric energy harvesters present a solution to the power requirements of many devices and can provide an alternative power source to batteries in a wide range of applications. For instance, the research community has been investigating their use in implantable and portable electric devices due to their high output power density and energy conversion efficiency, suitability for miniaturization, and CMOS compatibility [1]. Micromachined piezoelectric vibrational energy harvesters have been reported in many applications such as medical energy harvesting (e.g., cardiac pacemakers), automotive applications (e.g., tire pressure monitoring systems), industrial applications, military applications, wireless sensor nodes, and many others [2,3,4,5].

However, the scaling down of these harvesters implies various design challenges in order to maintain sufficient power output and well-suited resonance frequencies to match those of ambient vibration sources that, depending on application, can be optimal below 500 Hz. For this purpose, researchers have been looking for new ways in optimizing harvester designs [6,7]. In this work, the energy harvester designs presented operate over a range of resonant frequencies ranging from 2 to 5 kHz, and the effect of geometry variations on the resonant frequency of these devices is studied. This frequency range was selected as a trade-off between silicon area of the harvester designs and their resonant frequency in order to fabricate these devices in a commercial MEMS process. It is important to note that the results of this study can be scaled to lower frequency ranges if larger size piezoelectric energy harvesters are considered. In this case, the analysis and optimizations presented here would still apply and enable more efficient designs.

Many studies have been performed on piezoelectric energy harvesting, and models have been presented, notably for the cantilever geometry [8,9]. Some studies have focused on the impact of the position and geometry of the proof mass on the resonance frequency; however, the mass has always been presented as a 3D proof mass placed on the tip of the harvester. In this work a different approach is presented, by considering the mass as a planar T-shaped cantilever. The main advantage with the mass being planar is the reduction of the device thickness and the simpler fabrication and assembly. This allows the use of commercial MEMS technologies which provide relatively precise control over the structure dimensions in order to ensure optimal performance and repeatability. In this work, a study on the effects of the proof mass geometry on the resonance frequency is presented in order to reduce it and achieve the maximum amount of harvested energy. Finite element methods (FEM) simulations have been performed and analyzed to study effect of the beam width to length ratio, proof mass area, and proof mass to cantilever mass ratio, based on the measurement results of fabricated prototypes.

This paper is structured as follows: first, the operating principle of a piezoelectric harvester with a proof mass is detailed and then FEM simulation results are presented, followed by an optimized T-shaped design, the process flow for the fabrication technology, measurement results, and a conclusion.

## 2. Operating Principle of a Piezoelectric Energy Harvester with a Proof Mass

**Figure 1:**
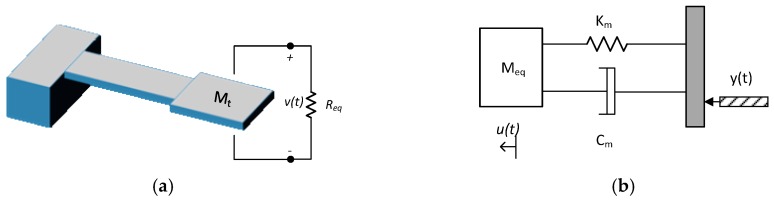
(**a**) Energy harvester; (**b**) mass-spring-damper model.

The MEMS piezoelectric energy harvesters studied here have the shape of a cantilever, clamped at one end and with a T-shaped proof mass attached at the free end, as depicted in Figure 1a. The piezoelectric layer is sandwiched between two electrode layers and excited such that the d31 piezoelectric coefficient is used, yielding an induced voltage across the electrodes in response to strain along the beam axis. The model of vibrational resonant structures is similar to the traditional mechanical resonator [10]. External accelerations stemming from vibrations are transmitted to a suspended mass causing a relative displacement. The material, geometry, and location of the proof mass affect the resonance mode and consequently the overall system performance. The lumped parameter model of a piezoelectric harvester would consist of a mechanical spring, *K*; an equivalent mass, *M*; and a damper, *C*, as shown in Figure 1b.

Inertia-based energy harvesters are reduced to a second-order spring-mass-damper system with equations based on Newton’s second law:

(1) $$f_{in}\left(t\right)=M_{eq}\cdot a\left(t\right)\;\mathrm{or}\;F_{in}\left(jw\right)=M_{eq}\cdot A\left(jw\right)$$

where *M_eq_* is the equivalent mass; *f_in_*, *a*, *F_in_*, and *A* are the force and acceleration in time and frequency domains, respectively. External vibrations of amplitude *y*(*t*) are transmitted to a suspended mass causing a relative displacement *u*(*t*). The harvester dynamics, based on the above equation to derive the mechanical domain equation with a single degree of freedom, can be represented by [11]:

(2) $$M_{eq}\ddot{u}\left(t\right)+C_{m}\dot{u}\left(t\right)+K_{m}u\left(t\right)-\theta v\left(t\right)=-M_{eq}\ddot{y}\left(t\right)\mu \;$$

where *K_m_* is the mechanical stiffness, *C_m_* is the mechanical damping, and *Ɵv* is the coupling force with *Ɵ* is the system coupling coefficient which is proportional to *d_31_*. Both *Ɵ* and *K_m_* depend on the geometry and strain distribution of the mode shape. All of these terms comprise mainly mechanical mode shapes and their derivatives [12]. Thus, by changing the mass, the mode shape is altered affecting all of these effective constants. The material, geometry, and location of a proof mass affect the modal analysis and consequently the analysis of the entire system. A correction factor *µ* has been added in Equation (2) to evaluate the effect of the mass. Its value ranges from 1 with a large tip mass to 1.566 with no tip mass [13]. When the center of gravity of the tip mass has an offset to the end of the piezo beam, an improved and detailed modelling of piezoelectric power harvesters with proof mass offset can be found in [14]. This can result in a more accurate expression of the mass matrix and dynamic force vector. This can provide a more practical design, which can avoid the use of material around the end of the piezoelectric length which can be damaged because of its brittle nature. In addition, note that the finite element equations proposed in [14] have been validated in [15].

**Figure 2:**
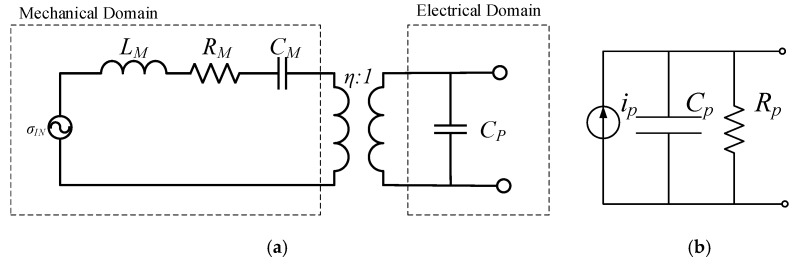
Piezoelectric harvester (**a**) coupled model and (**b**) uncoupled model [17].

The equivalent electrical circuit, shown in Figure 2a, can be seen as a mechanical spring mass system coupled to an electrical domain through a transformer that converts a strain to current. In the mechanical domain, the input stress is represented by *σ*, the mechanical mass by *L_M_*, the mechanical stiffness by *C_M_*, and the mechanical losses by *R_M_*. The piezoelectric coupling is modelled as a transformer, and *C_P_* represents the electrical domain plate capacitor composed by the piezoelectric material [16]. At resonance, the whole circuit can be transposed to the electrical domain. In the electrical domain, the cantilever-based piezoelectric harvester can be modelled as a current source in parallel with a parasitic capacitor and parasitic resistor, as illustrated in Figure 2b. Applying Kirchhoff circuit laws to the equivalent electrical circuit to determine the electrical domain equation yields:

(3) $$\theta \dot{u}\left(t\right)+C_{P}\dot{v}\left(t\right)+\frac{V\left(t\right)}{R_{eq}}=0$$

where $\theta \dot{u}$ is the current, *v* is the voltage, and *R_eq_* is the external load.

The natural frequency of a spring mass system, considering a stiffness K, is expressed as:

(4) $$\omega_{n}=\sqrt{\frac{K}{M_{eq}}}$$

where K varies depending on the structures. For a cantilever beam, $K=3EI/L^{3}$, where *E* is Young’s modulus of elasticity, *I* is the moment of inertia, and *L* is the length of the beam. *E* is the ratio of stress to strain while *I* depends on the beam width and thickness.

If the harvester is driven by a harmonic base excitation y(t)=Ysin(ωt), then the inertial mass *M_eq_* moves and the mechanical power which is to be converted to electrical power by the piezomaterial is given by [16]:

(5) $$P_{m}=\frac{M_{eq}\zeta Y^{2}{\left(\frac{\omega }{\omega_{n}}\right)}^{3}\omega^{3}}{{\left[1-{\left(\frac{\omega }{\omega_{n}}\right)}^{2}\right]}^{2}+{\left[2\zeta \left(\frac{\omega }{\omega_{n}}\right)\right]}^{2}}$$

where ζ is the damping ratio. For maximum energy conversion efficiency, the driving frequency of the harvester, ω, has to match its resonance frequency, $\omega_{n}$. The maximum output power occurs at $\omega =\omega_{n}$, and is given by [16]:

(6) $$P_{max}=\frac{M_{eq}Y^{2}\omega_{n}{}^{3}}{4\zeta }$$

Maximizing the power by operating at the natural frequency emphasizes the selection of piezomaterial and dimensions. The power delivered is proportional to the inertial mass. While the damping affects the Q-factor and bandwidth of the harvester, any variation in the excitation frequency results in a sharp drop-off in the power harvested. Note that Equations (5) and (6) do not consider the effect of the piezomaterial on the output power. This depends on the energy transduction of the strain across the piezomaterial. It has been shown in [16] that the output power depends on the strain experienced in the piezoelectric layer, its dimensions, and its piezoelectric coefficient.

## 3. Simulation Results

**Figure 3:**
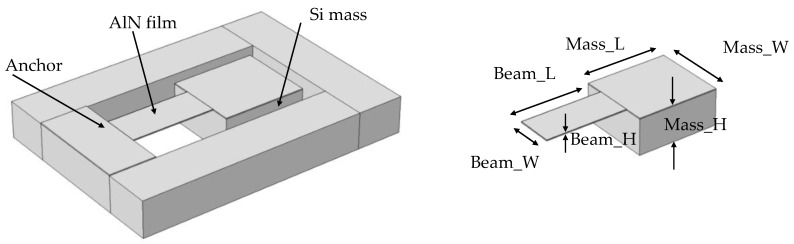
T-shaped harvester with the representation of the dimensions.

The MEMS energy harvester features a central beam that is connected to a proof mass. The design has six design degrees of freedom, namely, the beam length (Beam_L), the beam width (Beam_W), the beam height (Beam_H), the mass length (Mass_L), the mass width (Mass_W), and the mass height (Mass_H), as illustrated in Figure 3. When the beam height equals the mass height, a planar T-shape is achieved. Eigen frequency simulation through COMSOL Multiphysics was used to assess the effect of varying these variables on the device performance.

**Figure 4:**
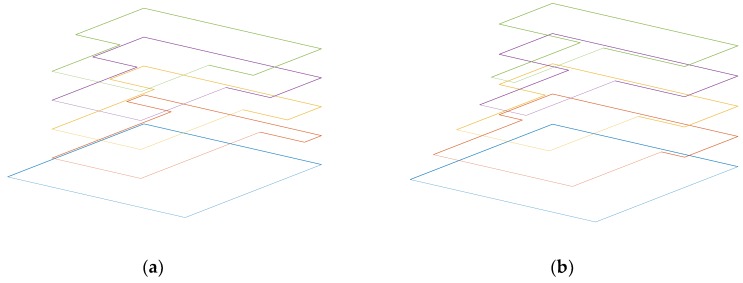
Overlay illustration of the variation of ratio of (**a**) the mass length (Mass_L) to the beam length (Beam_L) and (**b**) the mass width (Mass_W) to the beam width (Beam_W).

In order to get the most accurate results, a model has been created in COMSOL, in which a triangular swept mesh was used. In order to compare the results between experimental and simulation results, the beam was anchored to the surrounding silicon substrate and an air bubble around the device was used to take into consideration air damping effects. The structures were fabricated in a commercial MEMS technology: PiezoMUMPs from MEMSCAP. For this reason, the studies were limited to the structures that could be realized using this process. Therefore, a limitation on the values of the parameters, due to the fixed thicknesses of the materials used, has been considered. The value of Beam_H has been defined as 10 μm, and Mass_H can only take two different values 10 μm or 400 μm, the latter possible if the handle portion of the substrate is kept below the T-shaped mass. The piezoelectric material used is aluminum nitride (AlN). The design variations in the dimension ratios of the mass length to the beam length and the mass width to the beam width are illustrated in Figure 4.

### 3.1. The Effect of Beam_L and Mass_L over a Structure of a Fixed Length

**Figure 5:**
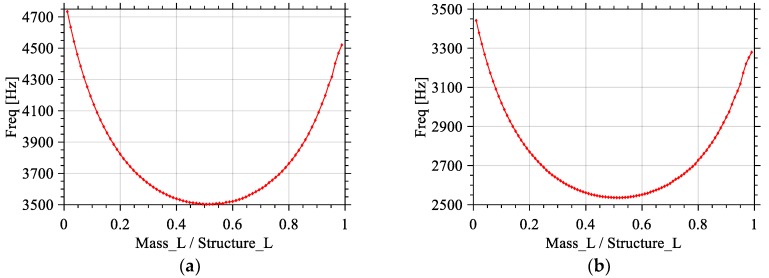
Effects of the variation of ratio of the mass length (Mass_L) to the total structure length (Structure_L) on the value of the first Eigen frequency for structure length of (**a**) 1700 μm and (**b**) 2000 μm.

In a set of simulations, the total length of the structure (Structure_L = Beam_L + Mass_L) and thickness (Beam_H and Mass_H) of the silicon structure are kept to 1700 μm and 10 μm, respectively. The length of the mass to length of the structure ratio (Mass_L/Structure_L) is varied from 0.01 to 0.98 by setting Mass_L from 20 to 1680 μm. Beam_W is selected to be half of Mass_W, which makes their values 300 μm and 600 μm, respectively. The total area of the proof mass (Mass_L × Mass_W) changes from 0.012 mm^2^ to 1.008 mm^2^, while the total area of the beam (Beam_L × Beam_W) changes from 0.504 mm^2^ to 0.006 mm^2^. This results in a ratio between the surface areas of the mass and the beam varying from 0.02 to 168, and the active area to available area ratio varying from 50.4% to 99.7%. These changes allowed for a reduction of 1.2 kHz on the value of the fundamental mode frequency as shown in Figure 5a.

Another set of simulations was carried-out with the following parameters: the total length of the structure (Structure_L) and thickness (Beam_H and Mass_H) of the silicon structure are kept to 2000 μm and 10 μm, respectively. The length of the mass to length of the structure ratio (Mass_L/(Mass_L + Beam_L)) changes from 0.01 to 0.99 by setting Mass_L from 20 to 1980 μm. Beam_W was chosen to be equal to half of Mass_W (i.e., 400 μm and 800 μm respectively). As a result, in that case, the total area of the proof mass (Mass_L × Mass_W) changed from 0.016 mm^2^ to 1.584 mm^2^, while the total area of the beam (Beam_L × Beam_W) changed from 0.792 mm^2^ to 0.008 mm^2^, therefore changing the surface mass to surface beam ratio from 0.02 to 198. Consequently, the ratio of surface used to available surface went from 50.5% to 99.5%. These changes allowed for a reduction of 0.9 kHz of the first Eigen frequency, as shown in Figure 5b.

Both these sets of simulations show that the lowest frequency is obtained when the length of the beam to length of the structure ratio is equal to 0.5. When the length of the beam is equal to the length of the mass, the best result is obtained. Moreover, the results are almost symmetric, e.g., if the length of the mass to length of the structure ratio is equal to 30% or 70%, similar results are attained.

The variation of the ratio of the length of the mass over the total length of the structure allows for a reduction in the resonant frequency of the design. However, this reduction depends on other parameters, and the effect of a variation of Beam_W and Mass_W was investigated as well.

### 3.2. The Effect of Beam_W/Mass_W

**Figure 6:**
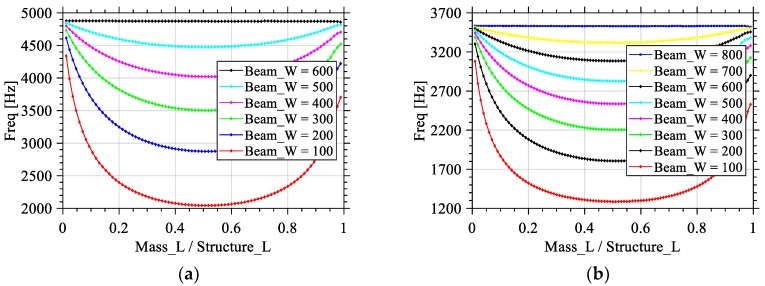
Effects of the variation of ratio of the mass length to the total structure length on the value of the first Eigen frequency for different Beam_W/Mass_W ratios, while conserving a total structure length of (**a**) 1700 μm and (**b**) 2000 μm.

In this set of simulations, the total length of the structure (Structure_L) and thickness (Beam_H and Mass_H) of the silicon structure are kept to 1700 μm and 10 μm, respectively. The length of the mass to length of the structure ratio (Mass_L/Structure_L) is varied from 0.01 to 0.98 by setting Mass_L from 20 to 1680 μm. Regarding the value of Mass_W, it was kept at 600 μm while using the value of Beam_W to explore different ratios for Beam_W/Mass_W such as 1/6, 1/3, 1/2, 2/3, 5/6, and 1, which results in Beam_W values of 100, 200, 300, 400, 500, and 600 μm, respectively. These different ratios allow a reduction of up to 2.2, 1.7, 1.2, 0.7, 0.3, and 0 kHz, respectively. The maximum reduction is reached when the length of the beam represents half of the total length of the structure for each given Beam_W, as seen in Figure 6a.

A second set of simulations was carried out with once again different values for the Beam_W/Mass_W ratio. The total length of the structure (Beam_L + Mass_L) and thickness (Beam_H and Mass_H) of the silicon structure are kept to 2000 μm and 10 μm, respectively. The length of the mass to length of the structure ratio (Mass_L/Mass_L + Beam_L) changes from 0.01 to 0.99 by setting Mass_L from 20 to 1980 μm. The value of Mass_W was chosen to be equal 800 μm while using the value of Beam_W is varied to explore different ratios for Beam_W/Mass_W such as 1/8, 1/4, 3/8, 1/2, 5/8, 3/4, 7/8, and 1, so respectively 100, 200, 300, 400, 500, 600, 700, and 800 μm. These different ratios allow a reduction of up to 1.7, 1.4, 1.1, 0.9, 0.6, 0.4, 0.2, and 0 kHz, respectively. The maximum of the reduction is reached when the length of the beam represents half of the total length of the structure, as seen in Figure 6b. Table 1 summarizes the above simulations as a percentage frequency reduction corresponding to beam width to mass width percentage.

According to the data presented, there is no optimal geometry stemming from reducing the beam width. However, while reducing the beam width allows for a reduction in the resonant frequency of the structure, this also reduces the surface available on which the piezoelectric material will be deposited, impacting output power. The structural integrity of the realized structure is also affected by reducing the beam width. Note that the targeted first out-of-plane flexural mode of the T-shaped device is of interest in this work. This mode is typically at a lower frequency than the torsional mode. However, the scaling down of the beam width can lower the torsional mode resonance frequency so that it overlaps or interferes with the targeted resonance operation. This should be considered in the device design when reducing the beam width, in addition to considering fracture and the reduced piezoelectric area.

When combining the two effects, it is noticed the change in frequency due to changing the Mass_L/Structure_L ratio is less significant when the width of the beam is near that of the mass, as seen in Figure 6.

### 3.3. Addition of a Proof Mass Using the Wafer Substrate

**Figure 7:**
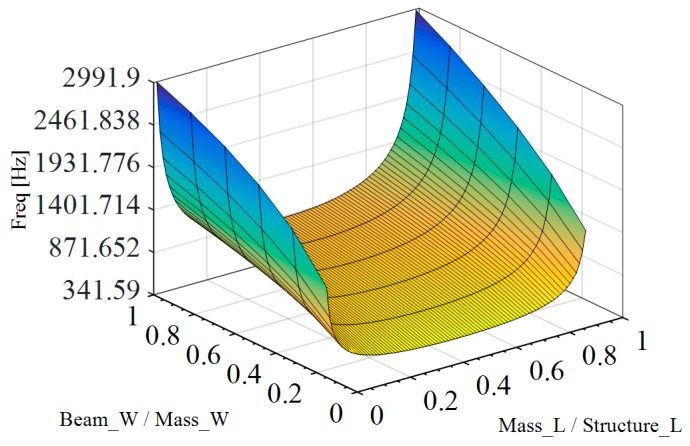
Effects of the variation of the Mass_L/Structure_L ratio on the value of the first Eigen frequency for different Beam_W/Mass_W ratios. Note that here the wafer substrate is used as a proof mass.

The impact of using the silicon wafer substrate as a proof mass to increase the mass depth was studied by performing similar simulations with a 400-μm-deep proof mass located under the mass portion of the structure. This allows a further reduction of the value of the resonant frequencies. As seen in Figure 7, a ratio of 0.5 between the length of the mass and the length of the structure still provides the best operating point. However, the change in frequency will be negligible from a Mass_L/Strucure_L ratio of 0.3 to 0.7 in comparison to the reduction in frequency observed when this ratio changes from 0 to 0.3 or from 1 to 0.7.

### 3.4. Addition of a Fixed-Area Mass

**Figure 8:**
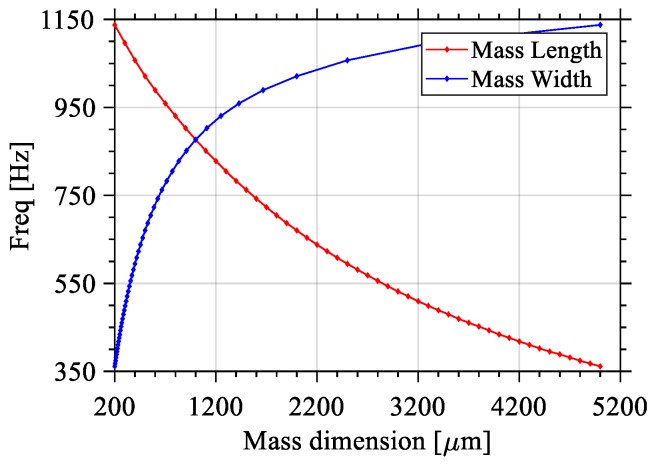
Effects of varying the length or the width of a constant-area (1 mm^2^) mass on the value of the first Eigen frequency of the structure.

If a fixed-area mass is to be added to the cantilever in order to lower the resonant frequency, then once again the mass geometry has to be studied carefully. For this set of simulations, the length (Beam_L), width (Beam_W) and thickness (Beam_H and Mass_H) of the silicon structure are set to 2000 μm, 500 μm, and 10 μm, respectively. To this beam, a mass is added, having a constant surface area of 1 mm^2^. Figure 8 shows the effect of adding a mass which will result in a range of possible frequencies depending on the mass geometry, namely mass length and width, while keeping height constant. When the mass length is longer, and consequently the mass width shorter, the resonance frequency is reduced. Note that, based on Figure 8, one can conclude that the addition of a proof mass of given area must be carried out by carefully selecting the mass geometry in addition to the beam geometry.

### 3.5. Discussion

As a result of the study of the effects of the geometry of the mass, the following conclusions are noted: (1) If the substrate is not used to increase the mass, a beam length that is equal to the mass length will yield the lowest resonant frequency; (2) If the substrate is added to the proof mass, then if the length of the mass is between 30 to 70% of the total structure length, the lowest resonant frequency will be attained; (3) In both cases, the ratio of the beam width over the mass width must be kept as small as possible in order to maintain a low resonant frequency, keeping in mind that the reduction of beam width will also reduce the harvested power, because of limited piezoelectric area; (4) Finally, care must be taken when considering a fixed mass geometry as this will result in a range of resonant frequencies (i.e., the longer its length, and consequently the shorter its width, the lower the resonant frequency).

## 4. T-shaped Optimized Design

Based on the conclusions reached from the simulation results, an optimized T-shaped design is proposed. To compare the effects of the change in mass geometry, a rectangular cantilever beam will be used as a comparison point. All the dimensions are presented in Table 2. The T-shaped design will follow the recommendations in considering a mass of the same thickness as the beam, a mass length equals to that of the beam, and a narrow beam width (within the laminations of the process technology). COMSOL frequency domain simulations were used to analyze output power as a function of the vibration frequency, with a given electrical load and the harmonic acceleration amplitude.

**Figure 9:**
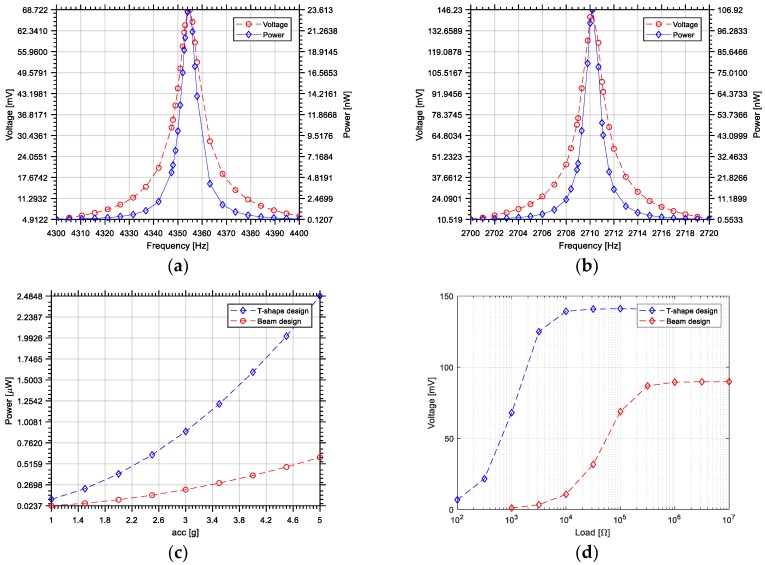
Simulation of the frequency response of the proposed design without using the substrate as a proof mass: (**a**) output voltage and power of the reference beam design; (**b**) output voltage and power of the T-shaped optimized design; (**c**) output power of the designs vs. acceleration; and (**d**) output voltage vs. resistive load value for both designs.

The first Eigen frequency of the T-structure is of 2710 Hz. The reference beam structure has a resonant frequency of 4350 Hz. Therefore, the proposed structure allows a reduction of 1640 Hz in resonant frequency. Figure 9a,b show the output voltage and power for both designs. Here, an acceleration of 1g along the z-axis and a resistive load of 100 kΩ are used in the simulations. The at-resonance voltage difference in the two designs of 69 mV (beam design) and 146 mV (T-shaped design) translates to an output power of 24 nW for the reference beam design to 107 nW for the optimized T-shaped design. The output power at resonance, for both designs versus the acceleration is depicted in Figure 9c, for a load of 100 kΩ. For instance, at an acceleration of 5g, the T-shaped design has an electric power output of about 2.5 µW compared to 0.5 µW for the cantilever, an improvement by a factor of 5. The output voltage as a function of the electrical load resistance at a harmonic acceleration amplitude of 1g at the resonant frequency of the devices is shown in Figure 9d. The T-shaped designs outputs more voltage at a given load and can sustain a smaller load resistance. This information can be used for the selection of a power conditioning circuit such that its load on the harvester is optimal.

While the T-shaped design proposed occupies smaller area than the reference beam design, it compares favorably and features various advantages, namely a lower resonant frequency, increased voltage and power output for a similar acceleration, and support for a smaller load resistance at a given acceleration. Accordingly, it is important to carefully dimension a cantilever-based energy harvester to ensure that the harvested power is optimal for a given available area.

## 5. Fabrication Process and Designs’ Dimensions

**Figure 10:**
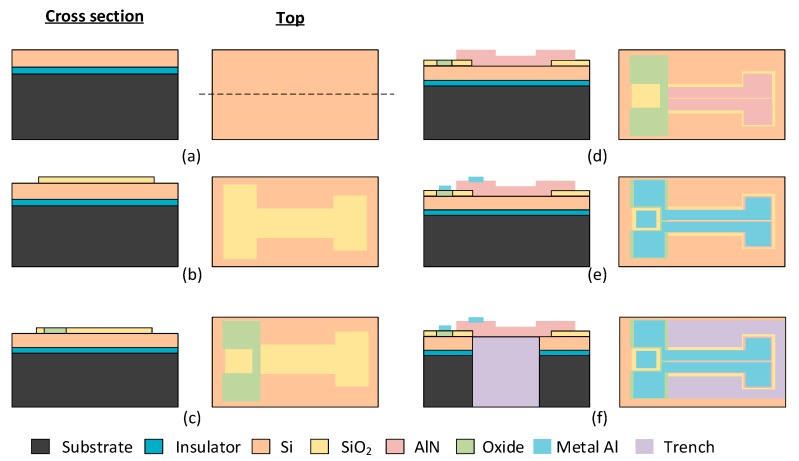
Overview of the processing steps of the PiezoMUMPs technology: (**a**–**f**) shows the process flow for the fabrication of a T-shape harvester.

The T-shape prototype designs were fabricated using the PiezoMUMPs process from MEMSCAP. PiezoMUMPs is a piezoelectric-based MEMS process that provides cost-effective access to MEMS prototyping. The fabrication process includes a 5-mask layer etching and patterning process briefly outlined in Figure 10 and has been detailed in [18]. The process is carried out on an N-type double-side polished silicon-on-insulator (SOI) wafer, used as the starting substrate (Figure 10a). First, the 10 µm silicon layer is doped using a phosphosilicate glass layer (PSG) that is deposited and then removed by wet etching to increase its conductivity. Then, a layer of silicon dioxide is patterned on the SOI wafer (Figure 10b). The silicon device layer is connected to the electrical ground and the 0.2 µm oxide isolates the signal pads from the ground plane (Figure 10c). A 0.5-µm-thick layer of piezoelectric aluminum nitride (AlN) is then deposited and patterned (Figure 10d). A 1.02 µm metal stack of 20 nm-thick chromium (Cr) for adhesion and 1 µm aluminum (Al) is deposited to form the electrical interconnects and the pads (Figure 10e). Lastly, the 400 µm substrate is etched from the backside to form release trenches (Figure 10f). Note that a portion of the substrate can remain below a given portion of the cantilever if the mask geometry is designed accordingly.

**Figure 11:**
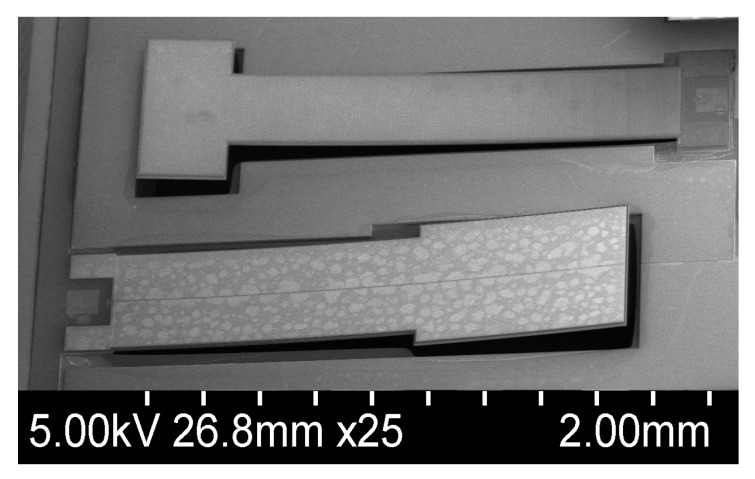
SEM micrograph of the fabricated harvesters Design 1 (bottom) and Design 2 (top).

The SEM micrographs of two of the fabricated devices, labelled Design 1 and Design 2, are shown in Figure 11. These fabricated designs were conceived in order to better characterize and model this vibrational piezoelectric energy harvester geometry. As such, the dimensions of the fabricated devices are presented in Table 3. In order to achieve higher energy outputs, interdigitated electrode designs have been proposed in the literature [19,20], in which an array of of narrow positive and negative electrodes are placed on the piezoelectric surface when it is fabricated. The fabricated structures are such that Design 2 is interdigitated while Design 1 is not. It can be noticed that the intrinsic stresses in the thin films are responsible for the out-of-plane upwards bending behaviour. This stems from the difference between the intrinsic stress of the deposited thin films and the crystalline silicon structure that has very low intrinsic stress. This deformation was not observed to have a significant impact on the resonant frequency of the devices characterized in this work, as measurements of identical structures showed a good stability of the resonance frequency between devices and a close match to the results was obtained in simulations.

Using FEM simulation, these devices have been recreated and simulated to extract their expected resonant frequencies and frequency response to a z-axis acceleration. The experimental data stemming from these designs allowed to tune the model in order to get a more accurate simulation of these designs. This was done by optimizing the material properties, anchoring, meshing, and damping of the FEM model. While the model allowed to get an accurate representation of the behavior of the system, the variations of the process accounted for a resonant frequency variation of up to 4.5%, if only Design 1 and Design 2 are considered. The value of that acceleration and of the damping of the surrounding air have been set to match the measurements made on the physically realized structures during the development of the model. This ensures that the extracted Q-factor from simulation matches that of the measurements.

## 6. Measurement Results

**Figure 12:**
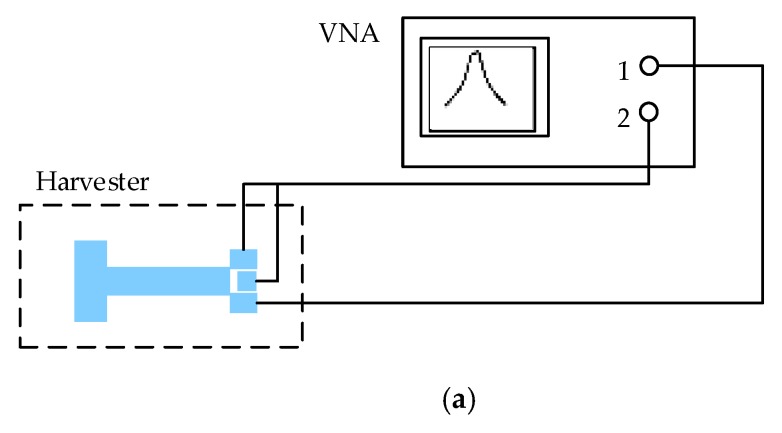

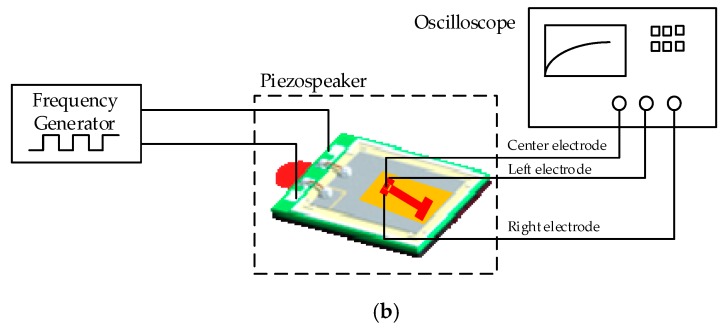
(**a**) Resonance test setup and (**b**) output power test setup.

**Figure 13:**
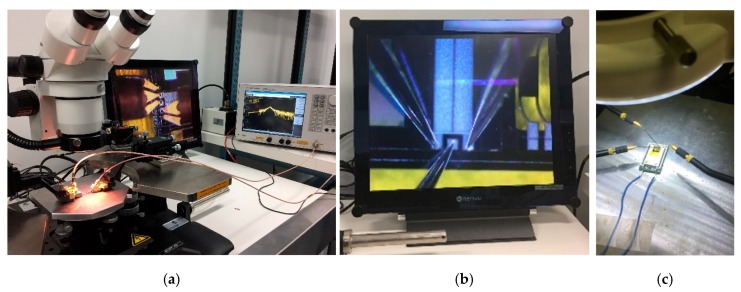
Photos of (**a**) the experimental test setup; (**b**) the device with the electrical probe tips; and (**c**) the piezospeaker setup with the device under test attached to it.

To test the fabricated prototype devices and acquire experimental data, the following process has been followed. The first step was to study the Eigen frequencies of the structures using COMSOL in order to gain insight into the expected resonance frequency of the devices. Then, using a vector network analyzer (VNA) (Model E5061B from Keysight, Santa Rosa, CA, USA), the values of the resonant frequencies were measured. The block diagram of the test setup is shown in Figure 12, while the experimental test setup is shown in Figure 13.

The two designs are such that the mass length is larger than the width in Design 1 while the mass width is larger than the length in Design 2. The average measured resonance frequency of Design 1 is of 4.6 kHz and that of Design 2 is 3.5 kHz. It is worth noting that the fabrication process is responsible for a small variation of the resonant frequencies of the devices from simulations, even after model tuning.

**Figure 14:**
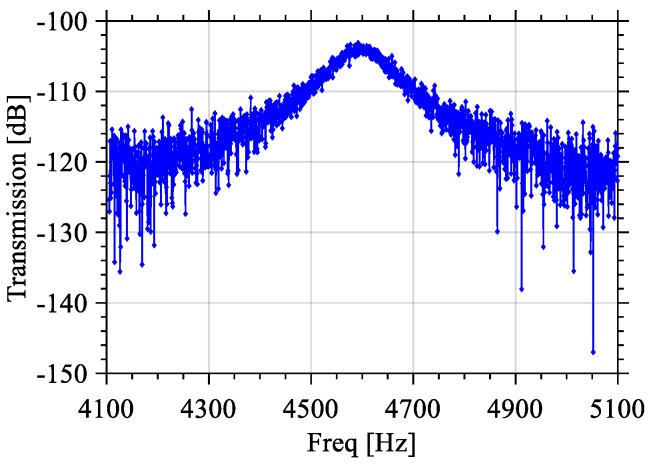
Measured frequency response of Design 1.

As mentioned earlier, Design 2 is interdigitated while Design 1 is not. However, in measurements no significant differences have been noticed, thus electrical tests focused only on Design 1. The output response of Design 1 measured with the VNA is shown in Figure 14, outlining the resonance frequency, bandwidth and the Q-factor. The bandwidth is measured to be about 63.3 Hz with a resonant frequency of 4.6 kHz and quality factor 72.7. The damping ratio is calculated to be ζ = 1/2Q = 0.007.

A piezo-speaker has been used to characterize the power output of this device providing a cost-effective method of testing. Piezo speaker APS2509S-T-R from PUI Audio (Dayton, OH, USA) was used [21], on which the devices were taped using kapton tape to generate the frequencies needed to measure their frequency response. This piezo speaker has a frequency range from 300 Hz to 20 kHz, therefore the tests were limited to excitation frequencies below 20 kHz. The mechanical motion of the piezo speaker is used here to vibrate the harvester device. In order to measure the voltages generated, probes connected directly to an oscilloscope without using an impedance matching circuit were used. The oscilloscope has been used as a load in this measurement.

**Figure 15:**
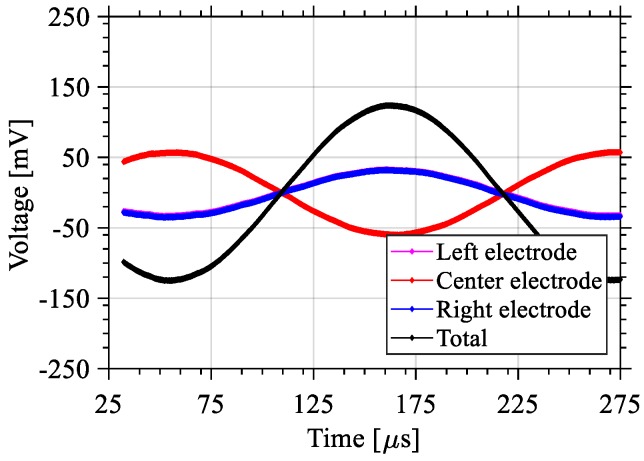
Output voltage of Design 1 in response to harmonic excitation at each electrode, along with the total combined output.

The response of the system when subjected to a sinusoidal excitation at a given frequency was characterized. An accelerometer from PCB Piezotronics (Depew, NY, USA) (model 352C65) was used to quantify the acceleration of the piezo speaker. The sinusoidal excitation of the piezo speaker has an amplitude of 10 V and a frequency 4350 Hz. This resulted in an acceleration 8.4g of the piezo speaker, and the generated power across the 1 MΩ oscilloscope load was measured to be 62 nW. The output voltage for the left, right, and center electrodes are shown in Figure 15. The left and right outputs expectedly show near identical responses. When combined, these outputs yield a maximum output voltage of 252 mV_p-p_.

**Figure 16:**
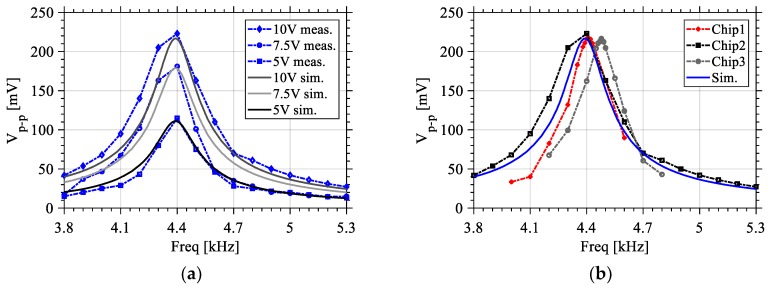
(**a**) Measured output voltage of Design 1 while varying the piezo speaker excitation voltage; and (**b**) variation between different dies of Design 1.

Figure 16a shows the output voltage corresponding to three input vibrations of different amplitudes and their corresponding simulations. As can be seen, the simulation results are in good agreement with the measurements. The piezo speaker used has a maximum input of 16 V_p-p_, however a limitation of 10 V max was imposed by the function generator used (Keysight 3320A function generator (Keysight, Santa Rosa, CA, USA)). The three input voltage amplitudes used on the piezo speaker are 5, 7.5, and 10 V. Figure 16b shows the generated voltages of three different dies of the same design outlining the process variation in terms of performance with an excitation voltage amplitude of 10 V with respect to simulation results, also showing a good correspondence with the measurements. Based on the measurement data received, the models had been revisited and optimizations had been performed for more accuracy in the fabrication of the prospective designs.

## 7. Conclusions

In this paper, a study on the effects of the geometry on piezoelectric energy harvester characteristics was presented. As discussed, improvements to the geometry of the harvester has an impact on the performance, in which dimension ratios have to be chosen carefully for optimal design performance. A study on T-shape harvesters has showed that the lowest frequency can be achieved when the length of the mass to the length of the structure ratio is 0.5, while keeping the beam width to mass width as small as possible.

An optimized T-shape design was proposed, and measurement results from prototypes used to validate the simulation model used to design the optimized T-shape device were presented. The optimized design stemming from the measured prototypes, simulation model, and study presented in this paper will be implemented for characterization and will then be integrated with a power conditioning circuit. While this study has allowed for device dimensions that lower the resonant frequency and increasing the output power, this can be further optimized by applying the technique to other beam geometries (e.g., [22]). Future work will investigate such structures in order to further increase the harvested power and lower the resonant frequencies within the 100 Hz to 1 kHz range.

## Figures and Tables

**Table 1:** Summary of the effect of the Beam_W/Mass_W ratio on the resonant frequency.

Beam_W/Mass_W (%)	12.5	25	37.5	50	62.5	75	87.5	100
**Resonant frequency reduction ^1^ (%)**	48	40	31	25	17	11	6	0

^1^ Beam_L = Mass_L.

**Table 2:** Proposed Design Parameters.

Design	L_B_ (μm)	W_B_ (μm)	H_B_ (μm)	L_M_ (μm)	W_M_ (μm)	H_M_ (μm)	Freq (Hz)	
Rectangular (reference)	900	800	10	900	800	10	4355	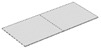
T-shaped (optimized)	900	300	10	900	800	10	2710	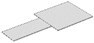

**Table 3:** Fabricated Design Parameters.

Design	L_B_ (μm)	W_B_ (μm)	H_B_ (μm)	L_M_ (μm)	W_M_ (μm)	H_M_ (μm)	Simulated Resonant Frequency (Hz)
Design 1	1000	400	10	700	500	10	4397
Design 2	1500	325	10	300	600	10	3591
